# The role of the Southern Ocean in the global climate response to carbon emissions

**DOI:** 10.1098/rsta.2022.0062

**Published:** 2023-06-26

**Authors:** Richard G. Williams, Paulo Ceppi, Vassil Roussenov, Anna Katavouta, Andrew J. S. Meijers

**Affiliations:** ^1^ Department of Earth, Ocean and Ecological Sciences, School of Environmental Sciences, University of Liverpool, Liverpool L69 3GP, UK; ^2^ Department of Physics, Imperial College London, London SW7 2AZ, UK; ^3^ National Oceanography Centre, Marine System Modelling, Proudman Building, Liverpool L69 3GP, UK; ^4^ British Antarctic Survey, Polar Oceans, Cambridge, UK

**Keywords:** heat uptake, carbon uptake, climate feedback, carbon feedback, transient climate response to carbon emissions, climate projections

## Abstract

The effect of the Southern Ocean on global climate change is assessed using Earth system model projections following an idealized 1% annual rise in atmospheric CO_2_. For this scenario, the Southern Ocean plays a significant role in sequestering heat and anthropogenic carbon, accounting for 40% ± 5% of heat uptake and 44% ± 2% of anthropogenic carbon uptake over the global ocean (with the Southern Ocean defined as south of 36°S). This Southern Ocean fraction of global heat uptake is however less than in historical scenarios with marked hemispheric contrasts in radiative forcing. For this idealized scenario, inter-model differences in global and Southern Ocean heat uptake are strongly affected by physical feedbacks, especially cloud feedbacks over the globe and surface albedo feedbacks from sea-ice loss in high latitudes, through the top-of-the-atmosphere energy balance. The ocean carbon response is similar in most models with carbon storage increasing from rising atmospheric CO_2_, but weakly decreasing from climate change with competing ventilation and biological contributions over the Southern Ocean. The Southern Ocean affects a global climate metric, the transient climate response to emissions, accounting for 28% of its thermal contribution through its physical climate feedbacks and heat uptake, and so affects inter-model differences in meeting warming targets.

This article is part of a discussion meeting issue 'Heat and carbon uptake in the Southern Ocean: the state of the art and future priorities'.

## 1. Introduction

The Southern Ocean plays a key role in the climate system by ventilating the ocean through the formation of mode, intermediate and bottom waters, and returning deep waters to the surface [1]. The ventilation over the Southern Ocean leads to anthropogenic carbon and heat being sequestered from the atmosphere, which is redistributed over the global ocean. The upwelling and mixing of deep waters to the surface over the Southern Ocean provides a unique window for the deep ocean to communicate with the atmosphere.

The different regimes for how the Southern Ocean interacts with the atmosphere are defined by the path of the eastward flowing Antarctic Circumpolar Current (ACC), driven by the prevailing westerly winds and surface buoyancy forcing, encircling Antarctica. The ACC shifts southeastward from its northernmost latitude in the Atlantic basin to its southernmost latitude in the eastern side of the Pacific basin. The upwelling follows an upward spiral on the southern flank of the ACC, concentrated where the flow interacts with topography [2]. Older, carbon-rich deep waters are upwelled and exposed to the atmosphere on the southern side of the ACC, while mode and intermediate waters are subducted on the northern side of the ACC, removing waters from being in contact with the atmosphere [3]. Accordingly, there are opposing air-sea carbon responses with an outgassing of CO_2_ from carbon-rich waters upwelled and mixed to the surface on the southward side of the ACC, and atmospheric CO_2_ drawn down by subduction of mode and intermediate waters to the north [4]. The subduction varies regionally according to the interaction of the surface circulation and the winter mixed layer, leading to localized regions of enhanced subduction wherever there is a downstream shoaling of the winter mixed layer [5].

The Southern Ocean is connected to the rest of the global ocean through large-scale flows involving the horizontal circulation, including the ACC and the neighbouring subtropical gyre circulations, and the vertical overturning circulation, including an upper cell and a bottom cell. The upper overturning cell is particularly important in redistributing anthropogenic carbon and heat, and is viewed in terms of a residual circulation, involving the sum of a wind-driven Ekman transport and the transport associated with mesoscale eddy circulation [6,7]. The residual circulation leads to a northward transport of surface waters and requires a buoyancy input from the atmosphere at a steady state [8–10]. This buoyancy input is provided by a combination of surface heat and freshwater fluxes including significant contributions from melting of sea ice [11]. The residual circulation carries these surface waters equatorward, so leading to a mismatch in the regions of air-sea heat uptake and ocean heat storage [12], as well as leading to a delay in the occurrence of climate warming in the Southern Ocean [13]. There is also a bottom cell driven by dense-water formation adjacent to the Antarctic continent and a return flow set by diapycnal mixing [14].

The Southern Ocean is disproportionately important in sequestering anthropogenic heat and carbon over the global ocean due to both the enhanced ventilation over the Southern Ocean and its transport of modified waters to the rest of the global ocean. This crucial role of the Southern Ocean is highlighted in analyses of a suite of Earth system models from the Coupled Model Intercomparison Project phase 5 (CMIP5) integrated over the historical period from year 1861 to 2005, revealing the Southern Ocean accounting for 43% ± 13% of the anthropogenic CO_2_ uptake and 75% ± 22% of the heat uptake by the ocean over the historical period [15], based upon the Southern Ocean being defined south of 30°S and occupying 30% of the global surface ocean area. This uptake of anthropogenic CO_2_ and heat exceeds their storage over the Southern Ocean due to the northward transport of anthropogenic CO_2_ and heat into the rest of the global ocean. There are additional CMIP5 model analyses of the Southern Ocean response [16] to historical and future forcing scenarios including assessing its heat uptake [17,18], circulation [19] and mixed-layer responses [18]. However, it remains unclear to what extent these analyses carry over for Coupled Model Intercomparison Project phase 6 (CMIP6) projections and the connection of the Southern Ocean to physical climate feedbacks, carbon-cycle feedbacks and to global climate metrics.

Here, we choose to examine the effect of the Southern Ocean on global climate metrics for CMIP6 model experiments following idealized scenarios with either a continuous or an abrupt increase in atmospheric CO_2_, rather than follow historical forcing scenarios. The advantage of the idealized scenarios is that by design the approach avoids the complications of non-CO_2_ greenhouse forcing, the response to aerosols and land-use changes, and so is standard for diagnosing climate metrics and feedbacks. Our analyses are though less relevant to understanding the historical response of the Southern Ocean given the strong hemispheric biases in historical radiative forcing [20], but are more relevant for future projections for the role of the Southern Ocean when the radiative forcing is expected to be more evenly distributed from increasing greenhouse gases [21].

A central global climate metric is how surface warming increases nearly linearly with the cumulative CO_2_ emission, called the transient climate response to emissions (TCRE) [22–27], which is defined in terms of the climate response to an idealized annual 1% increase in atmospheric CO_2_. This climate metric is related to how much carbon may be emitted before exceeding a warming target [28]. This climate metric is affected by how heat and carbon are sequestered, as well as by the physical and carbon feedbacks operating in the climate system [29]. The physical climate feedbacks in these Earth system models are diagnosed from an idealized abrupt 4×CO_2_ forcing scenario [30–32]. The carbon response of the Earth system models is diagnosed in terms of carbon-concentration and carbon-climate feedbacks [33,34] from model experiments with or without a climate response following a continuous annual rise in atmospheric CO_2_ [35–38].

In this study, the role of Southern Ocean on the global climate response to carbon emissions is explored by examining a combination of these heat, carbon and climate metrics. Analyses of the CMIP6 Earth system model experiments are performed following an idealized 1% annual increase in atmospheric CO_2_ over 150 years. The climate responses over the global ocean and the Southern Ocean are assessed with the Southern Ocean defined as the ocean extending south of 36°S. The methods to diagnose the climate response are described in §2, including the different subsets of the CMIP6 models employed for each of the diagnostics. The thermal response is examined in §3 extending from the surface warming response, the ocean uptake and storage of heat, to the top-of-the-atmosphere radiative balance and the resulting physical climate feedbacks. The carbon response is addressed in §4 including the storage and uptake of carbon, and its relationship to carbon-cycle feedbacks. The connection between the ocean heat and carbon uptake and physical climate feedbacks is then made to the global climate metric, the TCRE in §5. Finally, the wider implications of the study are discussed in §6.

## 2. Methods

The suite of models and diagnostic heat and carbon balances employed in this study are next described.

### (a) Subsets of CMIP6 models

The role of the Southern Ocean in determining the global climate response to carbon emissions is assessed following the CMIP6 experiments forced by an annual 1% rise in atmospheric CO_2_ integrated for up to 150 years. Different subsets of the CMIP6 models are used depending on the data archived and model experiments performed (table 1). The surface warming, radiative forcing, top-of-the-atmosphere heat balance and physical climate feedbacks are diagnosed using 21 CMIP6 models. The ocean heat uptake and storage is diagnosed using a smaller subset of 12 models. The ocean carbon uptake and carbon-cycle feedbacks are diagnosed using 11 models that have performed additional biogeochemical coupled model experiments with increasing atmospheric CO_2_ but excluding warming or climate change. The diagnostics of the global climate metric, the TCRE, are performed on 12 models that have archived the thermal and carbon data needed to diagnose its drivers.

**Table 1.** List of CMIP6 models used for the different diagnostics in this study: radiative forcing, planetary heat uptake and radiative response, and climate feedback; ocean heat content and air-sea heat flux; ocean carbon content and carbon feedback and the climate metric, the transient climate response to emissions.

CMIP6 model following a 1% annual increase in atmospheric CO_2_	radiative forcing and climate feedback diagnostics	ocean heat content and heat flux diagnostics	ocean carbon content and feedback diagnostics	transient climate response to emissions diagnostics	reference
ACCESS-CM2	y	y			Bi *et al.* [39]
ACCESS-ESM1-5	y	y	y	Y	Ziehn *et al.* [40]
BCC-ESM1	y				Wu *et al.* [41]
BCC-CSM2-MR	y			y	Wu *et al.* [42]
CanESM5	y	y	y	y	Swart *et al.* [43]
CanESM5-CanOE			y		
CESM2	y	y		y	Danabasoglu *et al.* [44]
CESM2-FV2		y			
CESM2-WACCM	y			y	
CESM2-WACCM-FV2		y			
CNRM-CM6-1	y	y			Voldoire *et al.* [45]
CNRM-ESM2-1	y	y	y	y	Séférian *et al.* [46]
GFDL-CM4	y				Held *et al.* [47]
GFDL-ESM4	y	y	y	Y	Dunne *et al.* [48]
GISS-E2-1-H	y				Kelley *et al.* [49]
HadGEM3-GC31-LL	y	y			Roberts *et al.* [50]
IPSL-CM6A-LR	y		y	y	Boucher *et al.* [51]
MIROCC6	y				Tatebe *et al.* [52]
MIROC-ES2L	y		y	y	Hajima *et al.* [53]
MPI-ESM1-2-LR	y		y	Y	Mauritsen *et al.* [54]
MPI-ESM1-2-HR		y			
MRI-ESM2-0	y		y		Yukimoto *et al.* [55]
NorESM2-LM	y		y	y	Seland *et al.* [56]
SAM0-UNICON	y				Park *et al.* [57]
UKESM1-0-LL	y	y	y	y	Sellar *et al.* [58]
number of CMIP6 models used for each analysis	21	12	11	12	

For all model variables, the underlying model drift is corrected for by subtracting the parallel pre-industrial control integration (piControl), where there is no increase in atmospheric CO_2_, from the model integrations initialized from the pre-industrial era and including a 1% annual increase in atmospheric CO_2_ (1pctCO_2_). The starting point of the piControl time series is the time used to initialize the 1pctCO_2_ run. The global means are calculated as grid cell area-weighted averages over the globe or over the region south of 36°S for the Southern Ocean.

The relationships for the radiative, heat and carbon balances used in this study are next described with the key terms listed in table 2.

**Table 2.** List of key physical, carbon and climate variables used in this study.

radiative budget and climate feedback terms		
ΔF	radiative forcing, expressed per unit horizontal area, positive directed into the climate system. Δ represents change since the pre-industrial	W m^−2^
N	heat uptake at the top of the atmosphere, expressed per unit horizontal area, positive represents an increase in heat uptake	W m^−2^
ΔR	radiative response, expressed per unit horizontal area, negative represents an energy flux out of the climate system into space	W m^−2^
λ	physical climate feedback parameter, positive represents a warming feedback acting to increase surface temperature	W m^−2^ K^−1^
$Q_{\mathrm{storage}}$	ocean heat storage over a defined volume evaluated from ocean heat content	J
$Q_{\mathrm{uptake}}$	ocean heat uptake over a defined volume, evaluated from a time-integral of air-sea heat flux directed into the ocean	J

carbon budget and carbon feedback terms		
$I_{\mathrm{em}}$	cumulative carbon emission, equivalent to the time-integral of emissions and evaluated from the change in carbon inventories in the atmosphere, land and ocean	PgC
$\Delta I_{a}$	change in atmospheric carbon inventory evaluated from atmospheric mixing ratio or atmospheric partial pressure of CO_2_	PgC
$\Delta I_{l}$	change in land carbon inventory over a defined volume	PgC
$\Delta I_{o}$	change in ocean carbon inventory over a defined volume, evaluated from ocean dissolved inorganic carbon	PgC
$\Delta I_{o,\mathrm{uptake}}$	change in ocean carbon inventory over a defined volume, evaluated from a time-integral of air-sea carbon flux directed into the ocean	PgC
$\Delta I_{\mathrm{CO}2}$	change in ocean carbon inventory linked to the rise in atmospheric CO_2_ used to define the carbon-concentration feedback, $\beta \Delta pCO_{2}$	PgC
$\Delta I_{\mathrm{climate}}$	change in ocean carbon inventory linked to the rise in global-mean surface temperature used to define the carbon-climate feedback, γΔT	PgC

the TCRE global climate metric and its contributions		
$\Delta T/I_{\mathrm{em}}$	transient climate response to emissions (TCRE), defined by the ratio of the change in global-mean surface temperature and the cumulative carbon emission	K EgC^−1^
ΔT/ΔF	thermal contribution to the TCRE depending on the physical climate feedback and the ratio of planetary heat uptake and radiative forcing, $-\lambda^{-1}(1-N/\Delta F)$	K (W m^−2^)^−1^
$\Delta F/\Delta I_{a}$	radiative contribution to the TCRE depending on the ratio of the radiative forcing and the change in the atmospheric carbon inventory	W m^−2 ^EgC^−1^
$\Delta I_{a}/I_{\mathrm{em}}$	airborne fraction from the ratio of the change in the atmospheric carbon inventory and the cumulative carbon emission, and related to the land-borne and ocean-borne fractions, $1-\Delta I_{l}/I_{\mathrm{em}}-\Delta I_{o}/I_{\mathrm{em}}.$ Carbon contribution to the TCRE	

### (b) Radiative diagnostics

The time-evolving top-of-the-atmosphere radiation budget is evaluated,

2.1 N(r,t)=ΔF(r,t)+ΔR(r,t),

where *r* and *t* denote space and time indices, *N* is the net heat input at the top of the atmosphere, Δ*F* is the increase in radiative forcing and Δ*R* is the radiative response, representing a change in radiative heat loss to space; all fluxes are in W m^−2^ and all positive values represent a planetary heat input. *N* is simply the net heat imbalance from the difference between the net absorbed shortwave radiation and the outgoing longwave radiation, directly obtained from monthly climate model output. To obtain the radiative forcing, Δ*F*(*r*,*t*), the radiative forcing is first estimated from a quadrupling of CO_2_, Δ*F*_4×CO2_(*r*), following the regression method of Gregory *et al*. [59] using abrupt-4 × CO_2_ data, applied at each grid point to obtain a spatial map. Second, at each grid point *r*, this radiative forcing Δ*F*_4×CO2_ is rescaled according to the time-evolving CO_2_ concentration in 1pctCO_2_, under the assumption of a logarithmic relationship between CO_2_ concentration and radiative forcing [60]:

2.2 $$\Delta F(r,t)=a(r)\ln(CO_{2}(t)/CO_{2}(t_{0})),$$

where *t*_0_ is the time of the pre-industrial era and *a*(*r*) = Δ*F*_4×CO2_(*r*)/ln(4).

From equation (2.1), the radiative response, Δ*R*(*r t*), is diagnosed once *N* and Δ*F* are known. The radiative response, Δ*R*(*r*,*t*), is interpreted in terms of the product of the physical climate feedback, *λ*(*r*,*t*) in W m^−2^ K^−1^ and the change in global-mean surface temperature, Δ*T*(*t*), such that

2.3 ΔR(r,t)=λ(r,t)ΔT(t).

The climate feedback parameter *λ*(*r*,*t*) is diagnosed following Gregory *et al*. [59], by taking the least-squares regression slope of Δ*R*(*r*,*t*) against Δ*T*(*t*) using annual-mean 1pctCO_2_ data. The climate feedback parameter is estimated at time *t* by regressing Δ*R*(*r*,*t*) against Δ*T*(*t*) over that time period from the start of the integration to time *t*. The temporal change Δ*λ*(*r*,*t*) is revealed by repeating the regressions over different time intervals with the same period, so *λ*(*r*,*t*) for years 75 and 150 are from regressions from year 1 to 75 and year 75 to 150, respectively.

To obtain further physical insight into the processes determining the value of the climate feedback, the physical climate feedback *λ*(*r*,*t*) is separated into contributions from changes in temperature (*T*), lapse rate (LR), relative humidity (RH), surface albedo (*α*) and clouds (*C*):

2.4 $$\lambda (r,t)=\lambda_{T}(r,t)+\lambda_{\mathrm{LR}}(r,t)+\lambda_{\mathrm{RH}}(r,t)+\lambda_{\alpha }(r,t)+\lambda_{C}(r,t).$$

This choice of decomposition minimizes the magnitude of large opposing-signed feedback contributions from temperature and water vapour [61], as compared with prior decompositions in terms of specific humidity. The decomposition in (2.4) is approximated by radiative kernels, following the approach of Soden *et al*. [62], which is widely used in the climate feedback community and applied in the analysis of CMIP6 models by Zelinka *et al*. [32].

### (c) Heat content and air-sea flux diagnostics

Ocean heat content, *Q*_storage_ in J, is evaluated over a volume integral,

2.5 $$Q_{\mathrm{storage}}(r,t)=\;\rho_{0}C_{p}^{\;}\int_{V}\theta (r,t)dV,$$

where *ρ*_0_ is the potential density (assumed here to be a constant value of 1024 kg m^−3^), *C_p_* is the specific heat capacity (assumed to be a constant of 4000 J kg^−1 ^K^−1^), *θ* is the ocean potential temperature, d*V* is the volume element and the integral is performed over the volume *V*. The heat content is calculated as an anomaly from the start of the 1% CO_2_ integration. The cumulative ocean heat uptake, *Q*_uptake_ in J, is calculated as

2.6 $$Q_{\mathrm{uptake}}(r,t)=\int_{t_{0}}^{t}\int_{A}H_{\mathrm{net}}(r,t)dAdt,$$

where $H_{\mathrm{net}}(r,t)$ is the surface net heat flux (positive into ocean) in W m^−2^ from all sources (CMIP6 variable *hfds*), and *A* is the ocean surface area. In practice care is needed, particularly near the poles, as in many models the ocean grids are extremely curvilinear, so that explicit cell surface area variable (*areacello*) is used to calculate cell areas and volumes. When integrated globally the cumulative ocean net surface heat flux should be identical to the tendency in the global ocean heat content anomaly. However, in several models (ACCESS-CM2, UKESM1-0-LL, CanESM5 and HadGEM3-GC31-LL), global ocean heat is not conserved with an offset of 5–18% between the temporal change in global ocean heat content and the area-integrated net surface flux; also reported in other studies (ACCESS-CM2, [63]) and in the UKESM1-0-LL (T Kuhlbrodt 2022, personal communication). The non-conservation of ocean heat is associated with a sea-ice related heat flux term being mistakenly excluded from the total ocean surface net heat flux variable (hfds). Inter-annual variability is still extremely well correlated between these variables and their net difference is small relative to inter-model variability, so these models have been retained in the ensemble.

### (d) Carbon content diagnostics

The change in the global ocean carbon inventory relative to the pre-industrial era, Δ*I_o_* in gC, is estimated from a volumetric integral of the dissolved inorganic carbon,

2.7 $$\Delta I_{o}(r,t)=c\int_{V}\Delta \mathrm{DIC}(r,t)dV,$$

where DIC is the dissolved inorganic carbon in molC m^−3^ and *c* = 12.01 g mol^−1^ is a converting factor from moles to grams of carbon and the integral is performed over the volume *V*.

The change in the carbon inventory Δ*I_o_* is separated into two contributions: (i) an inventory contribution Δ*I*_CO2_ from rising atmospheric CO_2_ involving a carbon-concentration *β* feedback and (ii) an inventory contribution Δ*I*_climate_ involving a carbon-climate feedback *γ* following the empirical relationship for the carbon-cycle feedbacks [33,35–38],

2.8 $$\Delta I_{o}(r,t)=\Delta \underset{\;}{I_{\mathrm{CO}2}(r,t)+\Delta I_{\mathrm{climate}}(r,t)=\underset{\Delta I_{\mathrm{CO}2}}{\underbrace{\beta (r,t)\Delta pCO_{2}(t)}}}+\underset{\Delta I_{\mathrm{climate}}}{\underbrace{\gamma (r,t)\Delta T(t)+\mathrm{non}\text{-}\mathrm{linear}\;\mathrm{terms}}},$$

where Δ*I_o_*(*r*,*t*) is the increase in the ocean carbon storage (in PgC) since the pre-industrial era, Δ*p*CO_2_(*t*) is the change in atmospheric CO_2_ (in ppm), Δ*T*(*t*) is the change in global-mean surface temperature (in K), *β*(*r*,*t*) is called the ocean carbon-concentration feedback parameter (in gC ppm^−1^) and *γ*(*r*,*t*) is the carbon-climate feedback parameter (in gC K^−1^).

The change in the ocean carbon inventory driven by the rising CO_2_, Δ*I*_CO2_, is estimated using the biogeochemically coupled simulation, and the change in the ocean carbon inventory driven by climate change, Δ*I*_climate_, from the difference between the fully coupled simulation minus the biogeochemically coupled simulation for the 1% yr^−1^ increasing atmospheric CO_2_, following the recommended C^4^MIP protocol of experiments [37,64].

The change in the ocean carbon inventory is further split into a preformed part, associated with the dissolved inorganic carbon transferred from the surface into the ocean interior due to the physical ventilation, and a regenerated part, associated with the dissolved inorganic carbon accumulated into the ocean interior due to biological regeneration of organic carbon [38,65], where the dissolved inorganic carbon is separated by

2.9 $$\mathrm{DIC}(r,t)=\mathrm{DI}C_{\mathrm{preformed}}(r,t)+\mathrm{DI}C_{\mathrm{regenerated}}(r,t),$$

with the DIC_regenerated_ estimated as

$$\mathrm{DI}C_{\mathrm{regenerated}}=R_{\mathrm{CO}}\Delta \mathrm{AOU}+\frac{1}{2}(\Delta \mathrm{Alk}-\Delta \mathrm{Al}k_{\mathrm{preformed}}-R_{\mathrm{NO}}\Delta \mathrm{AOU}),$$

where *R*_CO_ and *R*_NO_ are constant stoichiometric ratios, AOU is the apparent oxygen utilization, Alk is the alkalinity and Alk_preformed_ is the preformed alkalinity, such that Alk − Alk_preformed_ gives the contribution to alkalinity from biological calcification. The preformed alkalinity is estimated from a multiple linear regression using salinity and the conservative tracer PO [66], with the coefficients of this regression estimated based on the surface alkalinity, salinity, oxygen and phosphate in each of the Earth system models [38]. In high latitudes in regions of deep water formation, there is a significant disequilibrium of oxygen at the ocean surface [67]. Hence the use of AOU leads to an overestimation of the regenerated carbon in high latitudes in our study.

The cumulative ocean carbon uptake relative to the pre-industrial era, Δ*I_o_*_,uptake_ in gC, is estimated for an area integral of the cumulative air-sea carbon flux.

2.10 $$\Delta I_{o,\mathrm{uptake}}(r,t)=\int_{A}\int_{{\;}_{0}}^{t}F_{\mathrm{carbon}}(r,t)dtdA,$$

where $F_{\mathrm{carbon}}$ is the air-sea flux of carbon into the ocean in gC m^−2^ s^−1^ over a surface area *A* and *t*_0_ is the time of the pre-industrial era.

### (e) Framework to interpret the transient climate response to emissions climate metric

The TCRE climate metric is defined by the global-mean, surface warming response to cumulative CO_2_ emissions in K EgC^−1^ following a 1% annual increase in atmospheric CO_2_,

2.11 $$\mathrm{TCRE}(t)\equiv \frac{\Delta T(t)}{I_{\mathrm{em}}(t)},$$

where Δ is the change since the pre-industrial taken as year 1850, Δ*T*(*t*) is the global-mean change in surface air temperature (in K) and *I*_em_(*t*) is the cumulative CO_2_ emissions (in EgC) since the pre-industrial.

The TCRE is usually viewed as a product of two terms: (i) the change in global-mean air temperature divided by the change in the atmospheric carbon inventory, Δ*T*(*t*)/Δ*I_a_*(*t*), related to the transient climate response and (ii) the airborne fraction, Δ*I_a_*(*t*)/*I*_em_(*t*), given by the change in the atmospheric carbon inventory Δ*I_a_*(*t*) (in PgC) divided by the cumulative CO_2_ emissions [22,24–26,68].

To gain more mechanistic insight as to how the TCRE is controlled, the TCRE is instead connected to an identity involving three terms: (i) a thermal dependence on radiative forcing, defined by the change in temperature divided by the change in radiative forcing, Δ*F*(*t*); (ii) the radiative forcing dependence on atmospheric CO_2_; and (iii) the airborne fraction [27,29,69–71]:

2.12 $$\mathrm{TCRE}(t)\equiv \frac{\Delta T(t)}{I_{\mathrm{em}}(t)}=\underset{\mathrm{thermal}}{\underbrace{\left(\frac{\Delta T(t)}{\Delta F(t)}\right)}}\underset{\mathrm{radiative}}{\underbrace{\left(\frac{\Delta F(t)}{\Delta I_{a}(t)}\right)}}\underset{\mathrm{carbon}}{\underbrace{\left(\frac{\Delta I_{a}(t)}{I_{\mathrm{em}}(t)}\right)}}.$$

Henceforth, the TCRE is discussed in terms of this product of the thermal dependence, the radiative dependence between radiative forcing and atmospheric carbon, and the carbon dependence involving the airborne fraction.

#### (i) Thermal dependence of the transient climate response to emissions

The thermal dependence of the TCRE in (2.12) may be further understood from the empirical global radiative balance (2.1) [59,72]. The increase in radiative forcing, Δ*F*(*t*), drives an increase in planetary heat uptake, *N*(*t*), over the globe minus a radiative response, Δ*R*(*t*) = *λ*(*t*)Δ*T*(*t*), which is assumed to be equivalent to the product of the increase in global-mean surface air temperature, Δ*T*(*t*), and the climate feedback parameter, *λ*(*t*):

ΔF(t)=N(t)−λ(t)ΔT(t).

The thermal dependence in (2.10) of surface warming on radiative forcing, Δ*T*(*t*)/Δ*F*(*t*), is then given by the product of the negative inverse of the climate feedback parameter, − *λ*^−1^(*t*), and the planetary heat uptake divided by the radiative forcing, *N*(*t*)/Δ*F*(*t*),

2.13 $$\frac{\Delta T(t)}{\Delta F(t)}=-\frac{1}{\lambda (t)}\left(1-\frac{N(t)}{\Delta F(t)}\right),$$

where 1 − *N*(*t*)/Δ*F*(*t*) represents the fraction of the radiative forcing that escapes to space and effectively warms the surface, rather than the ocean interior.

#### (ii) Carbon dependence of the transient climate response to emissions

The carbon dependence of the TCRE in (2.12) involves the airborne fraction, Δ*I_a_*(*t*)/*I*_em_(*t*), and is related to the changes in the ocean-borne and land-borne fractions [73],

2.14 $$\frac{\Delta I_{a}(t)}{I_{\mathrm{em}}(t)}=1-\left(\frac{\Delta I_{o}(t)}{I_{\mathrm{em}}(t)}+\frac{\Delta I_{l}(t)}{I_{\mathrm{em}}(t)}\right),$$

where the changes in the ocean and land inventories are denoted by Δ*I_o_*(*t*) and Δ*I_l_*(*t*) (in PgC), respectively.

Hence, the controls of the TCRE may be identified using the framework (2.12) by combining with (2.13) and (2.14) as

2.15 $$\mathrm{TCRE}(t)\equiv \frac{\Delta T(t)}{I_{\mathrm{em}}(t)}=\underset{\mathrm{thermal}}{\underbrace{\frac{-1}{\lambda (t)}\left(1-\frac{N(t)}{\Delta F(t)}\right)}}\underset{\mathrm{radiative}}{\underbrace{\left(\frac{\Delta F(t)}{\Delta I_{a}(t)}\right)}}\underset{\mathrm{carbon}}{\underbrace{\left(1-\left(\frac{\Delta I_{o}(t)}{I_{\mathrm{em}}(t)}+\frac{\Delta I_{l}(t)}{I_{\mathrm{em}}(t)}\right)\right)}},$$

involving contributions from the climate feedback parameter, the ratio of the planetary heat uptake and the radiative forcing, the ratio of the radiative forcing and the change in atmospheric carbon, and the changes in the ocean-borne and land-borne fractions.

## 3. Thermal response

The thermal response of our subsets of CMIP6 models to an annual 1% rise in atmospheric CO_2_ is discussed in terms of the surface warming response, ocean heat storage and air-sea uptake over both the globe and the Southern Ocean. In order to understand the inter-model differences in the surface warming and ocean heat uptake, the radiative balance at the top of the atmosphere is discussed, including the planetary heat uptake and the radiative response, and the connection to physical climate feedbacks.

### (a) Surface warming response

**Figure 1.**
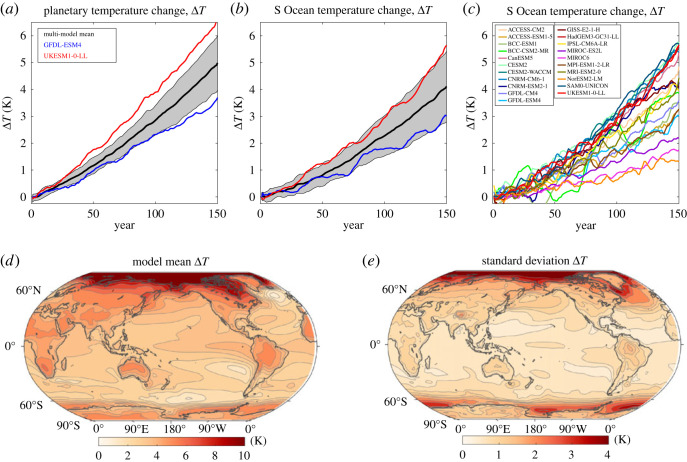
Increase in surface air temperature Δ*T* in K following the 1% yr^−1^ increasing atmospheric CO_2_ experiment: time series over (*a*) the globe and (*b*,*c*) the Southern Ocean including individual model responses together with global maps of (*d*) inter-model mean (with a global mean of 4.1 K) and (*e*) inter-model standard deviation for years 121–140. Diagnostics based upon 21 CMIP6 models. (Online version in colour.)

There is a progressive rise in global-mean surface air temperature in the suite of 21 models following the 1% increase in annual atmospheric CO_2_, their model mean increasing in their global average by 4.1°C by years 121–140 (figure 1*a*, black line). The rise in surface air temperature is slightly smaller over the Southern Ocean (defined by the ocean south of 36°S), reaching typically 3.3°C by years 121–140 (figure 1*b*, black line). The magnitude of the surface warming varies across the CMIP6 models (figure 1*a*,*b*, grey shading and figure 1*c*). For example, the global-mean surface warming of GFDL-ESM4 is only 3.0°C, while for UKESM1-0-LL it is 5.5°C by years 121–140; these values reduce slightly to 2.4°C and 4.3°C, respectively, over the Southern Ocean (figure 1*a*, blue and red lines).

The surface warming projections reveal most surface warming occurring over the northern high latitudes and over the land (figure 1*d*). The response varies over the Southern Ocean, changing from relatively weak surface warming between 36°S and 60°S, to enhanced surface warming between 60°S and Antarctica. The inter-model spread in surface warming is usually greatest in the same regions as where the surface warming is largest, particularly over the northern high latitudes and the southern latitudes of the Southern Ocean (figure 1*e*). There is also a wide inter-model spread over the subpolar North Atlantic, where there is limited surface warming in some models, and within the Atlantic to Indian sectors of the Southern Ocean.

Hence, the Southern Ocean stands out as a region of large inter-model spread in surface warming compared with the rest of the global ocean, even though the northern flank of the Southern Ocean only experiences relatively modest surface warming. The large inter-model spread, especially at high latitudes, may reflect the ongoing issues with sea surface, warm biases in the Southern Ocean [74]. These surface biases are believed to be at least partially related to cloud representation and consequent surface shortwave heating biases [75], and exacerbated by large inter-model differences in sea-ice retreat [76]. While advances have been made in cloud physics in CMIP6, there appears to be only partial success in reducing the magnitude of the surface warm biases in CMIP6 [77] compared with their larger magnitude in CMIP5 and CMIP3 [78].

### (b) Ocean heat uptake and storage

**Figure 2.**
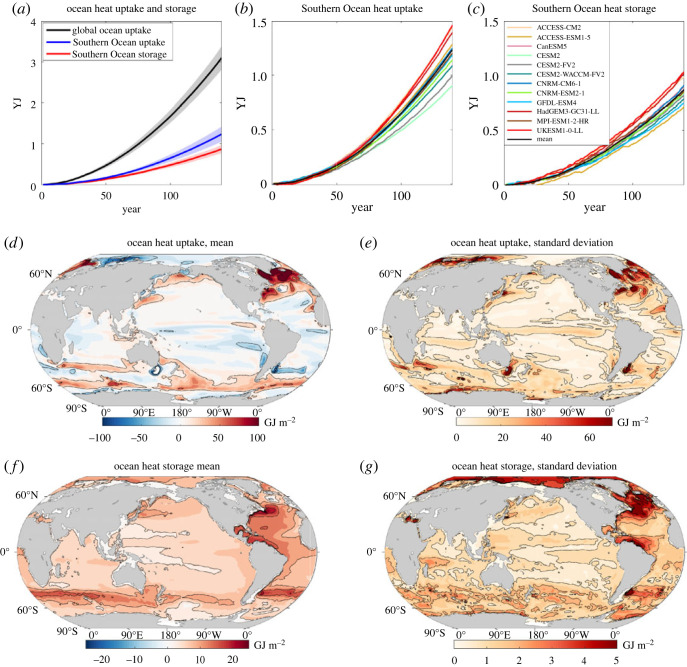
Global and Southern Ocean heat uptake and storage: (*a–c*) increase in ocean heat storage in 10^24^ J (or 1 YJ) over the global ocean (black line, grey shading) and over the Southern Ocean (blue) under the 1% yr^−1^ increasing atmospheric CO_2_ experiment, where the model mean is given by the full line and the shading represents 1 s.d. The Southern Ocean uptake of heat from the air-sea flux is included (blue) and exceeds the Southern Ocean storage of heat (red) due to the northward heat transport to the rest of the global ocean. Diagnostics based on 12 CMIP6 models and the individual responses of the GFDL-ESM4 (fine blue line) and UKESM1-0-LL (fine red line) are included. Maps for the model mean (*d*,*f*) and inter-model standard deviation (*e*,*h*) for time-accumulated ocean heat uptake (*d*,*e*) and ocean heat storage (*f*,*g*) are for a unit horizontal area, so in GJ m^−2^ for years 121–140; note the greater scale for (*d*) and (*e*). (Online version in colour.)

The increase in radiative forcing from the rise in atmospheric CO_2_ drives a rise in global ocean heat uptake of 2.74 ± 0.41 YJ (1 Yotta Joule ≡ 10^24^ J) by years 121–140 based on a model mean of 12 CMIP6 models (figure 2, black line). There is a large ocean heat uptake over the Southern Ocean, reaching 1.10 ± 0.16 YJ for the model mean by years 121–140 (figure 2*a*, blue line), accounting for 40% ± 5% of the global ocean rise in heat content.

There is an accompanying smaller rise in ocean heat storage over the Southern Ocean, typically 0.78 ± 0.9 YJ by years 121–140 (figure 2*a*, red line), which is 27% ± 2% of the increase of global ocean heat content, consistent with the Southern Ocean proportion of 27% of the global ocean volume. The Southern Ocean heat uptake exceeds this Southern Ocean heat storage with the excess heat transported northward to the rest of the global ocean. This northward heat transport out of the Southern Ocean reaches 0.32 ± 0.12 YJ and accounts for typically 29% of the heat uptake over the Southern Ocean.

#### (i) Pattern of ocean heat uptake and storage

Maps of the accumulated air to sea heat flux reveal a narrow band of enhanced ocean heat uptake along 60°S around the Southern Ocean, as well as over parts of the subtropical and subpolar gyres of the North Atlantic and North Pacific (figure 2*d*, red shading). There is enhanced ocean heat storage northward of the regions of ocean heat uptake in the Southern Ocean (figure 2*f*), consistent with how anthropogenic heat is taken up by the Southern Ocean and then accumulates at northern altitudes through the northward heat transport by the overturning circulation [12,13]. There is also an extensive accumulated storage of heat over the Atlantic basin, particularly over the subtropical gyres with their deep thermoclines. There is a larger inter-model spread in the ocean heat uptake and storage over the higher latitudes of both hemispheres and over the Atlantic basin (figure 2*e*,*g*).

#### (ii) Inter-model differences in the global and Southern Ocean response

**Figure 3.**
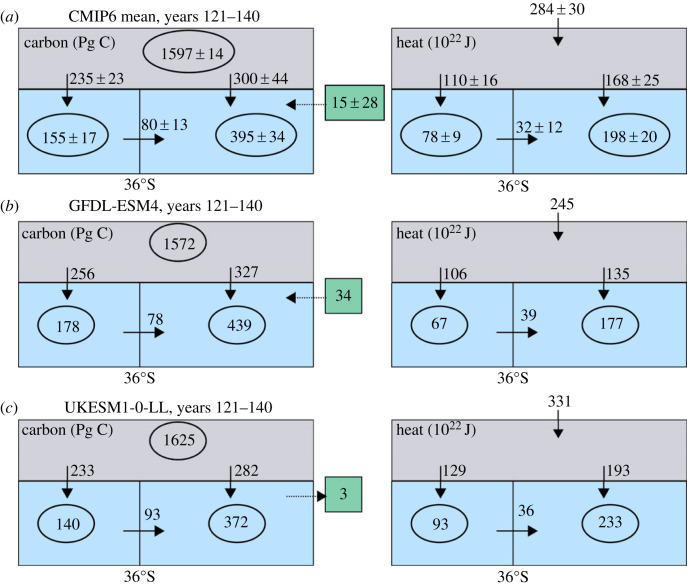
Decadal composites for the increase in carbon inventories in PgC (left column) and heat storage in 10^22^ J (right column) for (*a*) a mean of CMIP6 models under the 1% yr^−1^ increasing atmospheric CO_2_ experiment together with individual model examples for (*b*) GFDL-ESM4 and (*c*) UKESM1-0-LL for years 121–140. Diagnostics based on 11 CMIP6 models for the carbon synthesis and 12 CMIP6 models for the heat composite. The increase in the carbon and heat inventories are shown within the atmosphere (grey box) and the ocean (blue box) separated into the Southern Ocean (south of 36°S) and the rest of the global ocean. The vertical arrows represent the air-sea flux into the ocean, the heat input at the top of the atmosphere and the horizontal arrows represents the inferred northward ocean transport of heat and carbon out of the Southern Ocean. The dashed arrows with the green boxes represents the mismatch in the atmosphere–ocean carbon balance due to land-ocean fluxes, which is assumed to occur north of 36°S. (Online version in colour.)

There are inter-model differences in the global ocean heat uptake and storage (figure 3, right panels), such that models with either a relatively weak or strong surface warming response correspond to a relatively small or large ocean heat storage, respectively. For example, GFDL-ESM4 with a modest surface warming response has a relatively small increase in global ocean heat content of 2.41 YJ by years 121–140 (figure 3*b*), while UKESM1-0-LL with a strong surface warming response has a 38% larger increase in global ocean heat content of 3.22 YJ (figure 3*c*).

There are inter-model differences in the relative importance of the Southern Ocean for this global ocean response. For GFDL-ESM4, a slightly larger proportion of 44% of the global heat uptake occurs in the Southern Ocean and 37% of this Southern Ocean uptake is transported northward to the rest of the global ocean. For UKESM1-0-LL, 40% of the global heat uptake occurs in the Southern Ocean and of this uptake 28% is transported northward. The inter-model differences in the heat storage over the Southern Ocean cannot be simply explained by differences in northward heat transport. For example, the Southern Ocean heat storage is smaller in GFDL-ESM4, reaching 0.67 YJ, and is larger in UKESM1-0-LL, reaching 0.93 YJ by years 121–140, and this storage difference of 0.26 YJ exceeds the slight difference in their time-integrated northward heat transport of −0.03 YJ between both models (figure 3*b*,*c*).

Hence, the Southern Ocean is a key region in sequestering heat from the atmosphere, accounting for 40% ± 5% of the global ocean cumulative heat uptake and redistributing 29% of this uptake northwards. This Southern Ocean proportion of global heat uptake is significantly smaller than the 75% ± 22% for CMIP5 model analyses of the historical response [15]. This contrast in response is related to the simpler pattern in the radiative forcing of our purely CO_2_ driven scenario compared with the historical scenario including other radiative forcing agents; see Discussion for more details (and electronic supplementary material, table S1). There are significant inter-model differences in the heat uptake and storage integrated over the global ocean and Southern Ocean (figure 3). These inter-model differences in ocean heat storage are *not* explained by differences in the ocean redistribution of heat. To understand why individual Earth system models simulate lower or higher ocean heat uptake and storage (figures 2*b*,*c* and 3*b*,*c*), we need to turn to the radiative balance at the top of the atmosphere.

### (c) Top-of-the-atmosphere heat balance

The radiative heat balance at the top of the atmosphere is a key indicator of the underlying climate response for the planet. The increase in radiative forcing, Δ*F*, from the rise in atmospheric CO_2_ in (2.1) drives an increase in the planetary heat uptake, *N*, minus a radiative response, Δ*R*, representing the change in the heat loss to space, such that

ΔF(r,t)=N(r,t)−ΔR(r,t),

where all terms are evaluated in W m^−2^ as an area-integrated heat flux divided by the surface area and positive values represent a heat input to the climate system.

#### (i) Planetary response

**Figure 4.**
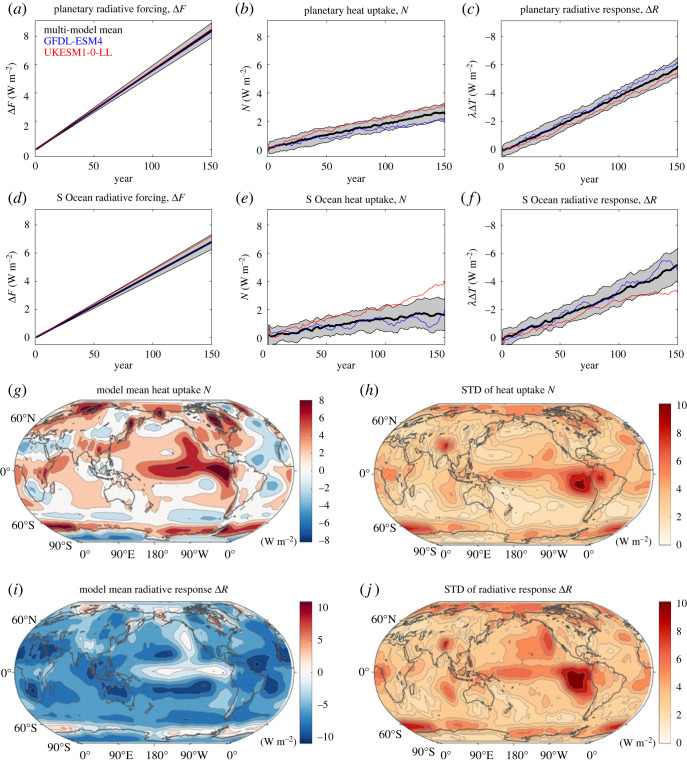
Time series for the heat balance at the top of the atmosphere over the globe (*a–c*) and the Southern Ocean (*d–f*) from 21 CMIP6 models following the 1% yr^−1^ increasing atmospheric CO_2_ experiment: (*a*,*d*) increase in radiative forcing, Δ*F*, (*b*,*e*) the planetary heat uptake, *N* and (*c*,*f*) the radiative response, Δ*R* (note the reversal in the sign of the *y*-axis), all in units of W m^−2^, where the area-integrated flux is divided by the surface area and positive values represent an input of heat. Global maps at the top of the atmosphere for (*g*,*h*) heat uptake, *N*, and (*i*,*j*) radiative response Δ*R* for the model mean (*g*,*i*) and standard deviation (*h*,*j*). A positive *N* and a positive Δ*R* represent a planetary heat input. Diagnostics for years 121–140. (Online version in colour.)

The planetary radiative forcing, Δ*F*, increases in time for all the models, reaching 7.3 W m^−2^ by years 121–140 over the globe (figure 4*a*), partitioned between an increase in planetary heat uptake, *N*, of 2.3 W m^−2^ and a radiative heat loss to space, Δ*R*, of −5.0 W m^−2^ by years 121–140 based on the mean of 21 models (figure 4*b*,*c*). There are inter-model differences in how the radiative forcing is partitioned. For a model with a modest surface warming response, GFDL-ESM4, there is a relatively low or modest planetary heat uptake and instead a high radiative heat loss to space (figure 4*b*,*c*, blue lines). By contrast, for a model with a strong surface warming response, UKESM1-0-LL, there is a relatively large planetary heat uptake and a low radiative heat loss to space (figure 4*b*,*c*, red lines).

The increase in the global top-of-the-atmosphere heat input, *N*, is almost entirely transferred via air-sea heat fluxes into an increase in global ocean heat content, accounting for 98% of the planetary heat uptake (figure 3*a*).

#### (ii) Southern ocean response

Over the Southern Ocean, the radiative forcing is slightly less than the global average, reaching 5.9 W m^−2^ for a model mean (figure 4*d*). There is less heat uptake and radiative heat loss compared with the global mean, reaching 1.6 and −4.3 W m^−2^, respectively, by years 121–140 (figure 4*e*,*f*). However, there is a larger inter-model spread over the Southern Ocean for both the top-of-the-atmosphere heat uptake and the radiative response, compared with the global mean (figure 4*e*,*f*, grey shading). The individual model responses differ over the global mean and the Southern Ocean, such as GFDL-ESM4 having relatively modest heat uptakes of 1.8 and 1.1 W m^−2^, while UKESM1-0-LL has relatively high heat uptakes of 2.8 and 3.2 W m^−2^, respectively (figure 4*a–f*, blue and red lines).

#### (iii) Pattern of heat uptake and radiative response at the top of the atmosphere

There is a heterogeneous pattern in the heat uptake at the top of the atmosphere from the mean of 21 models with an enhanced uptake varying regionally from 6 to 8 W m^−2^ after years 121–140 over the equatorial and eastern Pacific Ocean, the Southern Ocean close to 60°S, and the western side of the North Atlantic and North Pacific subtropical gyres (figure 4*g*, red regions). Conversely, there are regions of less heat uptake over the northern flank of the Southern Ocean and much of the central Atlantic basin (figure 4*g*, blue regions). The inter-model spread is largest in those regions of enhanced heat uptake, particularly over the equatorial and eastern Pacific and the Southern Ocean, south of 60°S (figure 4*h*).

The radiative response is generally negative and reveals enhanced heat loss to space over much of the subtropics and mid-latitudes (figure 4*i*, blue regions). However, there are localized regions where the radiative response is positive and so enhances the heat input at the top of the atmosphere over the equatorial and eastern Pacific and the southern flank of the Southern Ocean, south of 60°S (figure 4*i*, red regions). The largest inter-model spread in the heat input at the top of the atmosphere and the radiative heat loss is over the eastern and equatorial Pacific, much of the Southern Ocean and the Arctic (figure 4*h*,*j*).

#### (iv) Contrasting patterns in heat uptake for the ocean and at the top of the atmosphere

**Figure 5.**
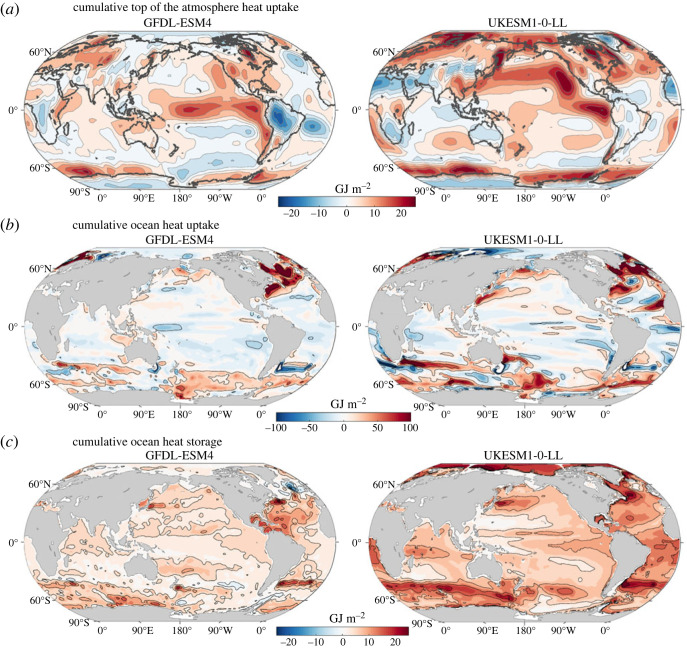
Global maps for (*a*) the cumulative top of the atmosphere heat uptake (GJ m^−2^), (*b*) the cumulative air-sea heat uptake (GJ m^−2^) and (*c*) the cumulative ocean heat storage (GJ m^−2^) for GFDL-ESM4 (left column) and UKESM1-0-LL (right column). A positive cumulative heat uptake represents a heat input into the system. Note the much larger range for the ocean heat uptake as opposed to the top of the atmosphere heat uptake and the ocean heat storage. The cumulative uptake is evaluated from time integrals of the heat flux from the start of the integration at year 0 until years 121–140. (Online version in colour.)

The planetary heat uptake *N*(*t*) at the top of the atmosphere is nearly entirely accounted for by the increase in heat storage over the global ocean. However, the spatial pattern in the heat uptake at the top of the atmosphere (figure 4*g*) is not the same as the pattern of cumulative ocean heat uptake over the globe (figure 2*d*), owing to the effect of horizontal redistribution of energy by the atmosphere; see time-cumulative heat responses for GFDL-ESM4 and UKESM1-0-LL in figure 5*a*,*b*. The top-of-the-atmosphere heat uptake reveals regions of high heat uptake over the tropics, especially over the central and eastern Pacific basin, which is not revealed in the maps of cumulative heat uptake by the ocean. There are bands of cumulative ocean heat uptake (figure 2*d*) and enhanced top-of-the-atmosphere heat uptake (figure 4*g*) over parts of the Southern Ocean, which may be possibly coincident with each other around 60°S.

There are inter-model differences in the patterns of the cumulative heat uptake at the top of the atmosphere and the cumulative heat flux and heat storage into the ocean (figure 5). The GFDL-ESM4 and UKESM1-0-LL have some similar patterns to each other in the cumulative heat uptake at the top of the atmosphere by years 121–140, with enhanced uptake over the tropical Pacific and the Southern Ocean (figure 5*a*), but there is a greater magnitude of heat uptake for UKESM1-0-LL and with more heat input over the North Pacific and the Arctic. The corresponding maps for cumulative ocean heat uptake for both models exhibit common features, with enhanced ocean heat uptake over a band in the Southern Ocean and over the North Atlantic, but more positive values for UKESM1-0-LL (figure 5*b*). There is also much more extensive cumulative ocean heat storage for UKESM1-0-LL, compared with GFDL-ESM4 (figure 5*c*).

In summary, the ocean is the key repository of the extra heat supplied to the climate system, accounting for typically 98% of the heat supplied at the top of the atmosphere. The pattern of the cumulative top-of-the-atmosphere heat flux is not generally the same as the pattern of the cumulative ocean heat uptake, although there are some bands of enhanced heat uptake over parts of the Southern Ocean. The top-of-the-atmosphere heat flux affects whether individual Earth system models experience relatively weak or high ocean heat storage, including the response over the Southern Ocean.

### (d) Physical climate feedbacks

The local radiative response, Δ*R*, in (2.1) is interpreted in terms of the product of the physical climate feedback, *λ*(*r*,*t*), and the change in global-mean surface temperature, Δ*T*, in (2.3), such that

ΔR(r,t)=λ(r,t)ΔT(t),

where a planetary heat loss, represented by a negative radiative response, Δ*R*, corresponds to a negative physical climate feedback parameter, *λ*. In this notation, a physical climate feedback reinforcing surface warming is represented by a positive value, while a physical climate feedback opposing surface warming is represented by a negative value.

#### (i) Global climate feedback and its key components

**Figure 6.**
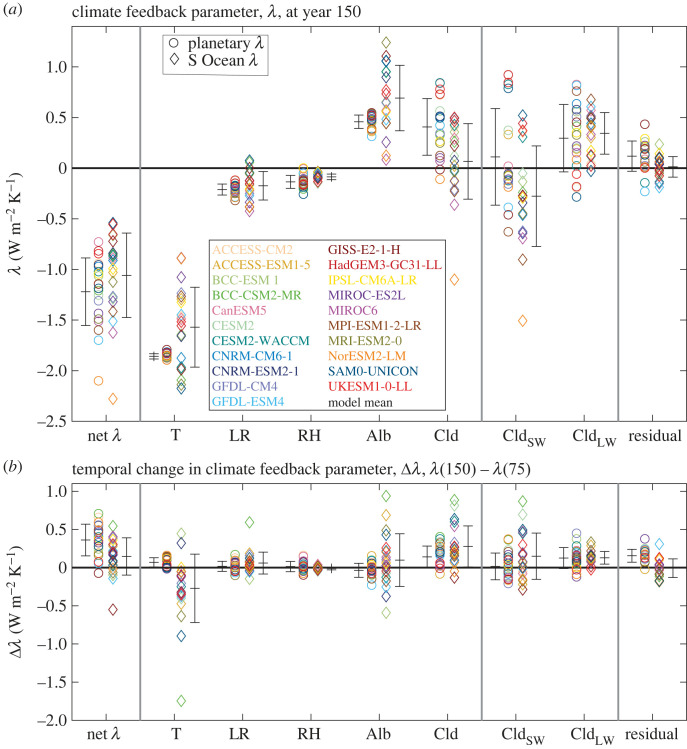
The physical climate feedback parameter, *λ* in W m^−2^ K^−1^, over the globe (circles) and the Southern Ocean (diamonds) at (*a*) year 150 and (*b*) the change from year 75 to year 150 (with each regression performed on the previous 75 years) from 21 CMIP6 models following the 1% yr^−1^ increasing atmospheric CO_2_ experiment. A more positive *λ* represents a positive feedback and less heat loss to space. The net climate feedback parameter *λ* is separated into individual components: the vertically uniform temperature, lapse rate, relative humidity, the surface albedo and the cloud effect on shortwave and longwave radiation. The residual represents the errors in defining the separation of physical climate feedback contributions. (Online version in colour.)

The physical climate feedback *λ* diagnosed over the globe provides an overall cooling with a model mean of −1.22 W m^−2^ K^−1^ at year 150 and a standard deviation of 0.33 W m^−2^ K^−1^ (figure 6*a*, circles). The physical feedback becomes slightly less negative in time by of 0.36 W m^−2^ K^−1^ from year 75 to 150 for the model mean, representing a weakening in the heat lost to space. This temporal change in the physical feedback is consistent with prior findings from coupled climate models forced with increasing CO_2_ concentration [30].

In order to interpret the model differences in physical climate feedback, the climate feedback is separated into components in (2.3) from the Planck temperature response to vertically uniform warming, *λ_T_*, the lapse rate response, *λ*_LR_, the relative humidity response, *λ*_RH_, the surface albedo response, *λ_α_* and the cloud response, *λ_C_*, made up of a shortwave and longwave cloud response in (2.4) following Held & Shell [61] (figure 6*a*, circles; table 3*a*). The physical climate feedback decomposition is estimated using radiative kernels and leads to a residual error of 0.12 W m^−2^ K^−1^ for a model mean, but can reach a magnitude of 0.4 W m^−2^ K^−1^ for individual models. Our following estimates of the contributions to climate feedback are consistent with the CMIP6 model mean estimates and inter-model spread reported by Zelinka *et al*. [32].

**Table 3.** The physical climate feedback parameter λ (W m^−2^ K^−1^) with positive values representing a positive feedback and reduced heat loss to space, while negative values represent a negative feedback providing an enhanced heat loss to space. Diagnostics over the globe and over the Southern Ocean based on a regression between the radiative response and global-mean surface temperature anomaly from 21 CMIP6 models.

	net	Planck	lapse rate	relative humidity	albedo	cloud	shortwave cloud	longwave cloud	residual
(a) physical climate feedback contributions from a regression over years 1–150									
planetary model mean, $\bar{x}$	−1.22	−1.86	−0.21	−0.14	0.46	0.41	0.11	0.30	0.12
standard deviation, $\sigma_{x}$	0.33	0.03	0.05	0.06	−0.07	0.28	0.48	0.33	0.15
Southern Ocean model mean, $\bar{x}$	−1.06	−1.57	−0.17	−0.09	0.62	0.07	−0.28	0.34	0.01
standard deviation, $\sigma_{x}$	0.42	0.39	0.14	0.03	0.32	0.37	0.50	0.21	0.10

(b) change in physical climate feedback contributions from a regression over years 76–150 minus years 1–75									
planetary model mean, $\bar{x}$	0.36	0.07	0.02	0.01	−0.04	0.14	0.02	0.13	0.16
standard deviation, $\sigma_{x}$	0.21	0.06	0.07	0.07	0.09	0.14	0.17	0.14	0.08
Southern Ocean model mean, $\bar{x}$	0.15	−0.27	0.06	−0.01	0.10	0.28	0.15	0.13	0.01
standard deviation, $\sigma_{x}$	0.24	0.45	0.14	0.02	0.35	0.27	0.30	0.08	0.12

The dominant contribution to the climate feedback is the Planck contribution of −1.9 W m^−2^ K^−1^ in this decomposition, providing a heat loss to space, for the model mean at year 150 (figure 6*a*). The corresponding lapse rate and relative humidity contributions provide contributions of −0.21 and −0.14 W m^−2^ K^−1^, again enhancing the heat loss to space. The corresponding surface albedo contribution is 0.46 W m^−2^ K^−1^ from a loss of snow and ice, which provides a positive feedback and a surface warming contribution. The cloud contribution is made up of shortwave and longwave contributions, providing a net cloud feedback of 0.41 W m^−2^ K^−1^ for the model mean at year 150, representing an overall warming contribution over the globe. The sign of the shortwave contribution varies across the individual models, primarily owing to model-dependent changes in highly reflective marine low cloud amount [32]; the model mean is 0.11 W m^−2^ K^−1^ providing a slight warming, but there is a large standard deviation of 0.48 W m^−2^ K^−1^. The longwave contribution generally provides a surface warming from a reduced emission of longwave radiation to space from rising cloud tops [79]; the model mean is 0.30 W m^−2^ K^−1^, providing a warming, and again there is a large standard deviation across the models of 0.33 W m^−2^ K^−1^.

The global climate feedback parameter increases in time by 0.36 W m^−2^ K^−1^ from years 75 to 150 for a model mean, acting to provide more surface warming (figure 6*b*, table 3*b*). This increase is mainly due to net changes in cloud feedbacks, involving both shortwave and longwave contributions. This increase is again consistent with the known time evolution of climate feedbacks in coupled climate models [30].

#### (ii) Climate feedback over the Southern Ocean and its key components

The climate feedback over the Southern Ocean in (2.3) is evaluated here in terms of the radiative response divided by the change in global-mean surface temperature rather than the change in local surface temperature; this choice offers a more straightforward interpretation of the contribution of the regional Southern Ocean feedback towards the global feedback [80]. The climate feedback over the Southern Ocean is slightly more positive for the model mean at year 150, reaching −1.06 W m^−2^ K^−1^ compared with the global-mean feedback of −1.22 W m^−2^ K^−1^, so both acting to oppose surface warming (figure 6*a*, diamonds; table 3*a*). There is a large inter-model spread in the Southern Ocean feedback with a standard deviation across the models of 0.42 W m^−2^ K^−1^.

The contributions for the physical climate feedback over the Southern Ocean include a surface warming contribution of 0.62 W m^−2^ K^−1^ from surface albedo, involving a loss of snow and sea ice, and of 0.34 W m^−2^ K^−1^ from longwave cloud involving enhanced downward radiative emission, versus an opposing surface cooling contribution of −0.28 W m^−2^ K^−1^ from increased shortwave cloud opacity [81] (figure 6*a*, diamonds). This overall positive contribution towards the physical climate feedback over the Southern Ocean from surface albedo and the net effect of clouds acts to enhance Southern Ocean surface warming and heat uptake.

There is a large inter-model spread for nearly all the feedback contributions over the Southern Ocean, particularly including large standard deviations for shortwave cloud, longwave cloud and surface albedo contributions of 0.50, 0.21 and 0.32 W m^−2^ K^−1^, respectively, at year 150.

The temporal change in the climate feedback over the Southern Ocean from years 75 to 150 features a model mean that becomes slightly more positive by 0.15 W m^−2^ K^−1^, so enhancing surface warming (table 3*b*). This weakening in the physical feedback leading to less cooling is due to reinforcing warming contributions from the surface albedo, shortwave and longwave cloud components of 0.10, 0.15 and 0.13 W m^−2^ K^−1^, respectively, which exceed the enhanced cooling contribution from the Planck feedback of −0.27 W m^−2^ K^−1^. There is a large inter-model spread in the change in the climate feedback over the Southern Ocean (figure 6*b*, diamonds) through inter-model differences in the Planck feedback (depending on the ratio of local to global warming), the surface albedo and the shortwave and longwave cloud contributions.

#### (iii) Regional pattern of the climate feedback and its key drivers

**Figure 7.**
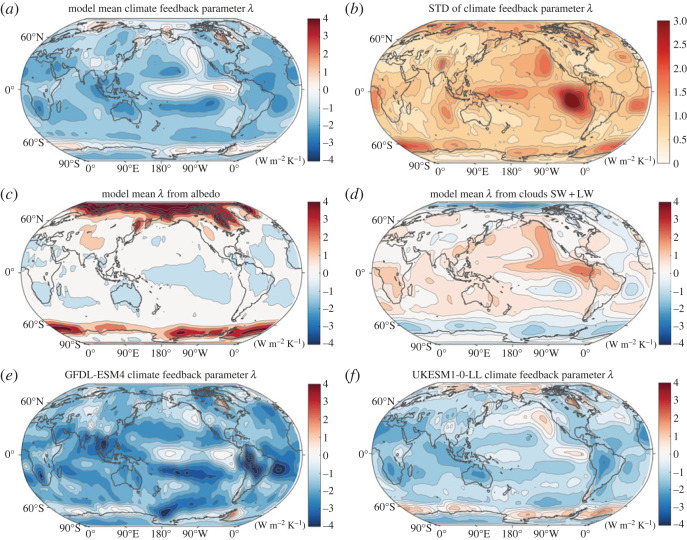
Global maps of the physical climate feedback parameter, *λ* in W m^−2^ K^−1^ for years 1–150 showing (*a*) the model mean and (*b*) the standard deviation of the net feedback together with the model mean components for (*c*) surface albedo and (*d*) net cloud feedbacks from shortwave and longwave. The physical climate feedback parameter is included for the individual Earth system models (*e*) GFDL-ESM4 and (*f*) UKESM1-0-LL. In (*a*) and (*c*) to (*f*), a physical feedback that promotes surface warming is shaded in red. (Online version in colour.)

A map of the model mean of the climate feedback parameter at year 150 reveals generally positive values representing a cooling feedback, and localized negative regions over the Southern Ocean and the tropical Pacific representing a warming contribution (figure 7*a*), following the pattern of the radiative response. The inter-model spread is large over these regions of warming contribution (figure 7*b*). The decrease in surface albedo from a loss of snow and sea ice leads to a more positive contribution to the climate feedback over the Southern Ocean and the Arctic (figure 7*c*, red). By contrast, for the model mean, the changes in clouds lead to a more negative feedback providing cooling over the Southern Ocean and the Arctic (figure 7*d*, blue), and instead a more positive feedback enhancing surface warming over the tropical Pacific (figure 7*d*, red).

There are inter-model differences in the net climate feedback response with the GFDL-ESM4 model providing a general negative feedback inducing cooling (figure 7*e*), whereas the UKESM1-0-LL model includes positive feedbacks over the Southern Ocean, the Arctic and the eastern Pacific, then inducing warming [82] (figure 7*f*).

In summary, there is a wide inter-model spread in the physical climate feedback over the globe and the Southern Ocean. The largest contributions to this spread over the globe are due to the shortwave and longwave cloud contributions, while over the Southern Ocean the surface albedo and the shortwave and longwave cloud contributions are particularly variable. These model differences in feedbacks are then connected to the contrasting surface warming responses over the globe and the Southern Ocean. The physical feedbacks over the globe and the Southern Ocean become more positive in time, acting to reinforce surface warming, which over the Southern Ocean is mainly due to the reduction in surface albedo and is partly opposed by a cooling contribution from changes in clouds. There is though significant inter-model spread in the surface albedo response over the high latitudes and the cloud responses over the globe.

## 4. Carbon response

The carbon response for a subset of CMIP6 models to an annual 1% rise in atmospheric CO_2_ is addressed in terms of the carbon uptake and storage over the global ocean and the Southern Ocean, and then connected to carbon-cycle feedbacks, where the ocean carbon storage is separated into a carbon-concentration and carbon-climate feedbacks.

### (a) Carbon storage and uptake

**Figure 8.**
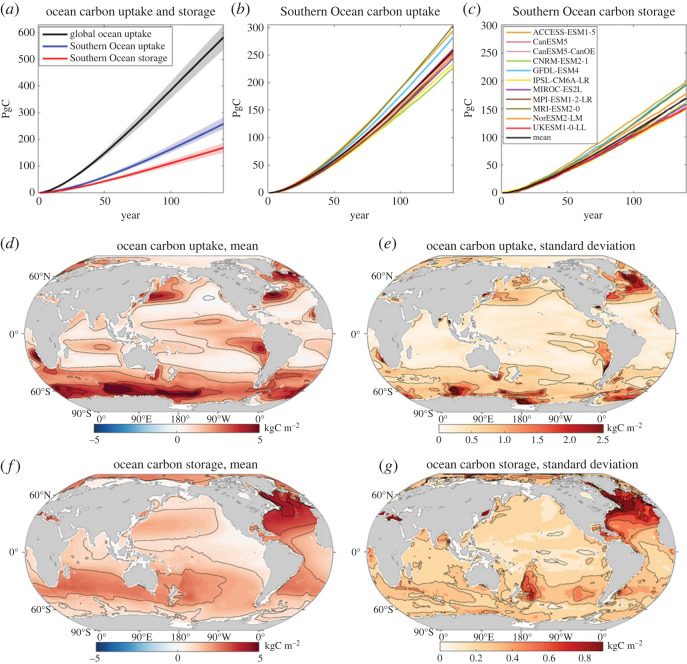
Increase in ocean carbon inventories in PgC: time series for (*a*) the global ocean (model mean is the black line, grey shading represents 1 s.d.) and the accumulated uptake and storage over the Southern Ocean (blue and red, respectively), and (*b*,*c*) the individual model responses for Southern Ocean uptake and storage. Global maps of the inter-model mean (*d*,*f*) and inter-model standard deviation (*e*,*g*) of the accumulated ocean carbon uptake and storage per unit area (kgC m^−2^) based upon years 121–140. The Southern Ocean uptake of carbon exceeds the Southern Ocean storage of carbon due to the transport of carbon northwards to the rest of the global ocean. Diagnostics based on 11 CMIP6 models under the 1% yr^−1^ increasing atmospheric CO_2_ experiment. (Online version in colour.)

The annual rise in atmospheric CO_2_ leads to an increase in the global ocean carbon inventory of 550 PgC by years 121–140 based on a mean of 11 CMIP6 models (figure 3*a*, left panel and figure 8*a*, black line). The Southern Ocean carbon storage reaches 155 PgC by years 121–140, so accounting for 28% of the global ocean storage (figure 8*a*, red line), close to how the Southern Ocean contributes 27% of the global ocean volume. The Southern Ocean carbon uptake is much greater than its storage, reaching 235 PgC by years 121–140, and accounts for 44% ± 2% of the global ocean carbon uptake (figures 3*a* and 8*a*, blue line). This excess in Southern Ocean carbon uptake is associated with a northward transport of carbon out of the Southern Ocean, redistributing 34% of the carbon uptake over the Southern Ocean to the rest of the global ocean (figure 3*a*).

There is a relatively limited inter-model spread for the Southern Ocean carbon uptake and storage (figure 8*b*,*c*), compared with the thermal response. For example, for GFDL-ESM4 and UKESM2-0-LL models with contrasting surface warming responses, there is a broadly similar carbon uptake over the Southern Ocean of 44% and 45% of the global uptake, respectively (figure 3*b*,*c*). However, there is a larger range in the proportions of Southern Ocean carbon uptake that is transported northward, which, for example, for GFDL-ESM4 and UKESM2-0-LL, varies from 30% to 40%, respectively (figure 3*b*,*c*).

#### (i) Pattern of ocean carbon uptake and storage

Maps of the cumulative carbon uptake for a mean of 11 models reveals high carbon uptake over the entire Southern Ocean (figure 8*d*). There is also enhanced carbon uptake over the extension of the Gulf Stream and Kuroshio currents on the western side of the subtropical gyres of the North Atlantic and North Pacific, most of the subpolar North Atlantic gyre and the eastern side of the equatorial Pacific Ocean. The model standard deviation in the cumulative carbon uptake is enhanced over these regions of high carbon uptake, particularly over the North Atlantic, both its subpolar gyre and the northern flank of the subtropical gyre, and over much of the Southern Ocean (figure 8*e*). The pattern of ocean carbon storage is more evenly distributed than that of the uptake with enhanced storage in the Atlantic basin and the mid-latitudes of the Indian and Pacific basins (figure 8*f*) and has larger inter-model variability over the North Atlantic (figure 8*g*).

Hence, the Southern Ocean is a key ocean region for sequestering carbon from the atmosphere, accounting for 44% ± 2% of the global uptake, and increasing the carbon content of the Southern Ocean, as well as redistributing typically 34% of this carbon uptake to the rest of the global ocean.

### (b) Carbon response and feedback

The ocean storage of carbon in these model projections include an inventory response dependent upon the rise in atmospheric CO_2_, referred to as the carbon-concentration feedback, and an inventory response dependent on the climate response, referred to as the carbon-climate feedback, represented by an empirical relationship (2.8),

$$\Delta I_{o}=\Delta \underset{\;}{I_{\mathrm{CO}2}+\Delta I_{\mathrm{climate}}=\beta (r,t)\Delta pCO_{2}(t)+\gamma (r,t)\Delta T(t)},$$

where Δ*I_o_* is the increase in the ocean carbon inventory (in PgC) since the pre-industrial, Δ*p*CO_2_ is the change in atmospheric CO_2_ (in ppm) and Δ*T* is the change in global-mean surface temperature (in K), *β*(*r*,*t*) is the ocean carbon-concentration feedback parameter (in gC ppm^−1^) and *γ*(*r*,*t*) is the carbon-climate feedback parameter (in gC K^−1^). The *β* and *γ* terms are usually referred to as feedbacks, but really should be viewed as representing the dependence of the change in the ocean carbon inventory on particular variables, rather than an amplifying or damping feedback.

#### (i) Ocean carbon response to rising atmospheric CO_2_

**Figure 9.**
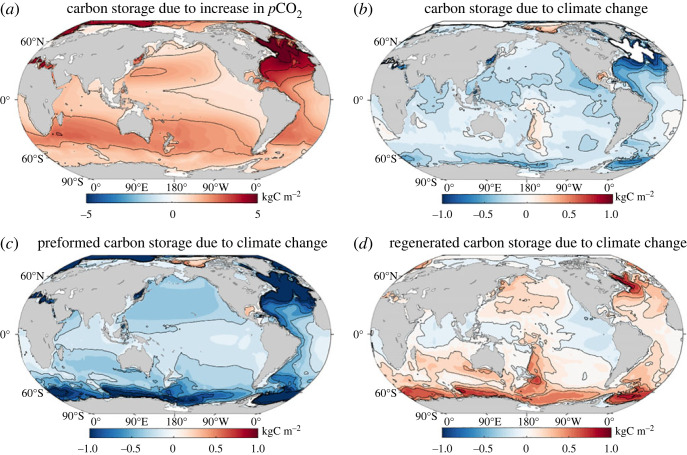
Maps of the regional contributions to the ocean carbon inventory based on a carbon-cycle framework: (*a*) carbon-concentration inventory change, *β*(*r*,*t*)Δ*p*CO_2_(*t*) (kgC m^−2^), and (*b*) carbon-climate inventory change, *γ*(*r*,*t*)Δ*T*(*t*) (kgC m^−2^), from a mean of 11 CMIP6 Earth system models under the 1% yr^−1^ increasing atmospheric CO_2_ experiment. Diagnostics are for years 121–140. The carbon-climate inventory change is separated into (*c*) a preformed component, depending on the preformed ocean carbon, and (*d*) a regenerated component, depending on the biologically regenerated ocean carbon. (Online version in colour.)

The ocean carbon inventory strongly increases through the rise in atmospheric CO_2_ with the carbonate chemistry acting to take up more carbon, so that *β* Δ*p*CO_2_ is always positive. As the ocean carbon inventory increases, there is an acidifying feedback effect that acts to inhibit ocean carbon uptake and enhance the fraction of carbon remaining in the atmosphere. However, this acidifying feedback is not sufficient to offset the ability of the ocean, via its carbonate chemistry, to hold more carbon with higher atmospheric CO_2_. A map of the change in the carbon storage due to the increase in atmospheric CO_2_, *β* Δ*p*CO_2_, reveals positive values across the global ocean with enhanced values over the subtropical gyres, particularly over the North Atlantic, and relatively weak values over the Southern Ocean (figure 9*a*). The carbon-concentration feedback *β* is nearly entirely controlled by its preformed component, *β*_preformed_, dependent on the carbon value in the mixed layer and its subsequent subduction into the ocean interior, and its regenerated component, *β*_regenerated_, dependent on how biology transfers carbon to depth in the ocean, is unimportant [38].

#### (ii) Ocean carbon response to climate change

Climate change weakens the ability of the ocean to absorb and store the additional carbon released in the atmosphere, so that *γ* Δ*T* is generally negative. This carbon-climate contribution *γ*Δ*T* is typically five times smaller than the carbon-concentration effect *β* Δ*p*CO_2_ (figure 9*a*,*b*), consistent with prior studies [35,83]. This weakening in the ability of the ocean to hold more carbon with climate change is associated with a warmer ocean acting to hold less carbon due to a solubility feedback and a more stratified ocean acting both to inhibit the ventilated transfer of carbon into the ocean interior and alter the biological drawdown of carbon. Consequently, a map of the regional contributions to changes in the carbon storage due to climate change, *γ*Δ*T*, reveals generally negative values (figure 9*b*).

The carbon-climate component, *γ*Δ*T*, is made up of a preformed contribution *γ*_preformed_Δ*T* that is strongly negative in the regions of strong ventilation over the Southern Ocean and North Atlantic (figure 9*c*), reflecting how climate change acts to inhibit the ventilated transfer of carbon into the ocean interior [84,85]. By contrast, the regenerated contribution, *γ*_regenerated_Δ*T*, is generally positive (figure 9*d*), indicating a greater carbon storage associated with the biological carbon pump due to climate change [35,86]. This feedback is likely dominated by an increase in the residence time of water at depth that enables an increase in the accumulation of regenerated carbon [38,85] and so the carbon held as dissolved inorganic in the ocean interior, rather than direct changes in the ocean biology.

Hence, the carbon-cycle diagnostics reveal the dominant contribution of the carbon-concentration effect, indicating a greater carbon uptake for higher values of atmospheric CO_2_ [35,86]. The Southern Ocean is an important region for the smaller carbon-climate feedback, with competing positive and negative contributions from the preformed and regenerated carbon [84,85], depending upon ventilation and residence times, respectively. Our diagnostics are based on the local vertical integrated carbon storage, but it should be noted that the carbon-cycle feedbacks can be further related to the formation and transport of water masses in the Southern Ocean [87].

## 5. Global climate metric, the transient climate response to emissions

The roles of the global ocean and Southern Ocean in sequestering heat and carbon, and their relation to the physical climate feedback and the carbon feedback, have been discussed. Now we turn to consider the ocean control of a global climate metric, the extent that surface warming increases with cumulative carbon emissions, referred to as the TCRE in (2.11),

$$\mathrm{TCRE}(t)=\frac{\Delta T(t)}{I_{\mathrm{em}}(t)},$$

where Δ is the change since the pre-industrial taken as year 1850, Δ*T*(*t*) is the global-mean change in surface air temperature (in K) and *I*_em_(*t*) is the cumulative CO_2_ emissions (in EgC).

This climate metric is defined in terms of the surface warming response to a cumulative carbon emission following an idealized scenario of a 1% annual increase in atmospheric CO_2_, as discussed here. This metric is important as the maximum permitted carbon emission before reaching a specified warming target is inversely proportional to the TCRE, such that models with a high TCRE have only a small permitted cumulative carbon emission before reaching a temperature threshold, while models with a smaller TCRE have a correspondingly larger permitted cumulative carbon emission.

**Figure 10.**
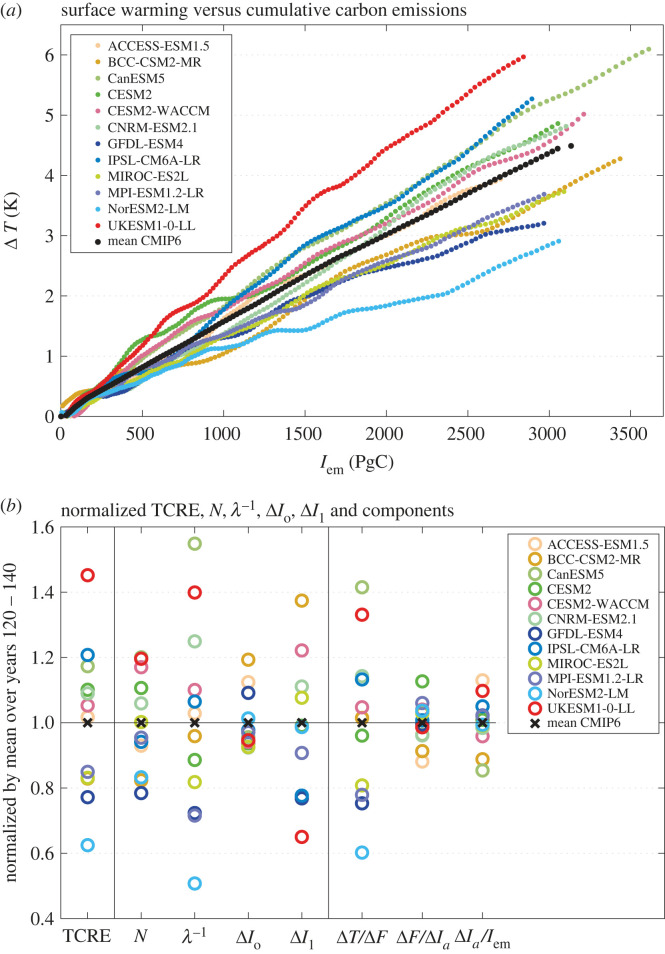
(*a*) Surface warming from the rise in global-mean, surface air temperature, Δ*T*(*t*) in K, versus cumulative carbon emissions, *I*_em_(*t*) in PgC, from 12 CMIP6 models following a 1% rise in atmospheric CO_2_ over 140 years. The slope of the lines defines the climate metric, the transient climate response to emissions, the TCRE. (*b*) Inter-model normalized spread for the TCRE for 12 CMIP6 models over years 121–140 defined by the individual model response divided by the mean of the models. This spread is included for the planetary heat uptake, *N*, the reciprocal of the planetary feedback parameter, *λ*^−1^, the change in the global ocean and land carbon storage, Δ*I_o_* and Δ*I_l_*. The normalized components of the TCRE are also shown: the thermal contribution, Δ*T*/Δ*F*, the radiative contribution, Δ*F*/Δ*I_a_*, and the carbon contribution, Δ*I_a_*/*I*_em_, where Δ*F* is the increase in radiative forcing and Δ*I_a_* is the increase in the atmospheric carbon inventory. (Online version in colour.)

For this subset of CMIP6 models, there is a nearly constant TCRE over the period of 140 years for each individual model, as indicated by the near constant slope in the surface warming when plotted against cumulative carbon emissions in figure 10*a*; the model mean of the TCRE for 12 CMIP6 models is 1.47 K EgC^−1^ with a standard deviation of 0.34 K EgC^−1^ at years 121–140 (table 4). There are though striking intermodal differences in the TCRE, as marked by the different slopes of the surface warming when plotted against the cumulative carbon emissions (figure 10*a*), with the normalized intermodal spread in TCRE ranging from 1.45 to 0.65 (figure 10*b*).

**Table 4.** Model mean, inter-model standard deviation and coefficient of variation for global-mean increase in (*a*) TCRE and its thermal, radiative and carbon contributions, (*b*) thermal terms and (*c*) carbon terms, based on years 121–140 for 12 CMIP6 Earth system models (used for the TCRE analysis) following a 1% annual increase in atmospheric CO_2_. The coefficient of variation is defined by the inter-model standard deviation divided by the model mean, evaluated at the same time. The coefficient of variation measures the inter-model spread and particularly large values greater than 0.1 are marked in italics, indicating a dominant contribution of that variable.

	TCRE	thermal contribution	radiative contribution	carbon contribution
(*a*) *TCRE and contributions*				
symbol	$\Delta T/I_{\mathrm{em}}$	ΔT/ΔF	$\Delta F/\Delta I_{a}$	$\Delta I_{a}/I_{\mathrm{em}}$
units	K EgC^−1^	K (W m^−2^)^−1^	W m^−2^ EgC^−1^	
model mean, $\bar{x}$	1.47	0.55	4.60	0.58
standard deviation, $\sigma_{x}$	0.34	0.13	0.30	0.05
coefficient of variation, $\sigma_{x}/\bar{x}$	*0.23*	*0.24*	0.07	0.08

	radiative forcing	planetary heat uptake	climate sensitivity	fraction of radiative forcing warming the ocean interior
(*b*) *thermal terms*				
symbol	F	N	*λ*^−1^	N/ΔF
units	W m^−2^	W m^−2^	K (W m^−2^)^−1^	
model mean, $\bar{x}$	7.32	2.31	−0.83	0.32
standard deviation, $\sigma_{x}$	0.47	0.34	0.25	0.05
coefficient of variation, $\sigma_{x}/\bar{x}$	0.06	*0.15*	*−0.30*	*0.14*

	cumulative carbon emission	airborne fraction	ocean-borne fraction	land-borne fraction
(*c*) *carbon terms*				
symbol	$I_{\mathrm{em}}$	$\Delta I_{a}/I_{\mathrm{em}}$	$\Delta I_{o}/I_{\mathrm{em}}$	$\Delta I_{l}/I_{\mathrm{em}}$
units	PgC			
model mean, $\bar{x}$	2747	0.58	0.19	0.22
standard deviation, $\sigma_{x}$	216	0.05	0.02	0.06
coefficient of variation, $\sigma_{x}/\bar{x}$	0.08	0.08	*0.12*	*0.29*

The question that we now wish to address is which processes are helping to determine inter-model spread in this climate metric (figure 10*a*,*b*) and then gain insight as to whether the Southern Ocean is playing a significant role in affecting this metric.

### (a) Controls of the transient climate response to emissions climate metric

To gain mechanistic insight as to how the TCRE is controlled, the TCRE is linked to an identity involving a thermal dependence on radiative forcing, defined by the change in temperature divided by the change in radiative forcing, Δ*F*(*t*) (in W m^−2^), and the radiative forcing dependence on atmospheric CO_2_ and a carbon dependence involving the airborne fraction, which may be expressed from (15) as

$$\mathrm{TCRE}(t)=\frac{\Delta T(t)}{I_{\mathrm{em}}(t)}=\underset{\mathrm{thermal}}{\underbrace{\frac{-1}{\lambda (t)}\left(1-\frac{N(t)}{\Delta F(t)}\right)}}\underset{\mathrm{radiative}}{\underbrace{\left(\frac{\Delta F(t)}{\Delta I_{a}(t)}\right)}}\underset{\mathrm{carbon}}{\underbrace{\left(1-\left(\frac{\Delta I_{o}(t)}{I_{\mathrm{em}}(t)}+\frac{\Delta I_{l}(t)}{I_{\mathrm{em}}(t)}\right)\right)}},$$

involving contributions from the climate feedback parameter, the ratio of the planetary heat uptake and the radiative forcing, the ratio of the radiative forcing and the change in atmospheric carbon, and the changes in the ocean-borne and land-borne fractions.

Inter-model differences in the TCRE, apparent in the different slopes in figure 10*a*, may be determined by any of the thermal, radiative and carbon variables in (2.15). A measure of the inter-model spread is provided by the coefficient of variation, defined by the inter-model standard deviation divided by the inter-model mean, and may be used to indicate the relative importance of different contributing terms. The coefficient of variation for the TCRE is 0.23 for years 121–140 (table 4*a*). In comparison, the coefficient of variations for the thermal, radiative and carbon contributions are 0.24, 0.07 and 0.08, respectively (table 4*a*), so that only the thermal contribution has a comparable coefficient of variation to that of the TCRE, suggesting that the thermal contribution provides the largest contribution to inter-model differences in the TCRE. As the TCRE is related to the product of these terms in (2.15), if the contributions were varying in a random manner, then the coefficient of variation for the TCRE is expected to be given by the root-mean square sum of the squares of each contribution, which would be 0.26, only slightly larger than the diagnosed value of 0.23. Hence, there is a slight partial compensation between these contributions. The central point remains that the dominant contribution to the inter-model spread for the TCRE for these CMIP6 models is from the thermal contribution and the contributions from the radiative and carbon contributions are much smaller on this centennial timescale; note that for a study of CMIP5 models, the intermodal spread is also strongly affected by the land carbon cycle [68].

#### (i) Thermal contribution to the transient climate response to emissions

The thermal contribution for the TCRE, Δ*T*/Δ*F*, is made up of contributions depending on the climate sensitivity, *λ*^−1^, and the fraction of radiative forcing warming the climate system, *N*/Δ*F*, in (2.15), which have coefficients of variation of 0.30 and 0.14, respectively (table 4*b*). The inter-model spread of the thermal contribution to the TCRE is then strongly affected by the climate sensitivity and by the fraction of radiative forcing used to warm the ocean; there is a partial compensation between these contributions as the coefficient of variation for Δ*T*/Δ*F* is mid-way between their values.

#### (ii) Carbon contribution to the transient climate response to emissions

The carbon contribution for the TCRE given by the airborne fraction, Δ*I_a_*/*I*_em_, in (2.15) for these CMIP6 models has a coefficient of variation of 0.08 and is made up of contributions from the ocean-borne and land-borne fractions with much larger coefficients of variation of 0.12 and 0.29 (table 4*c*), suggesting that there is significant uncertainty in components of the carbon cycle, but they are partially cancelling in their contribution to the airborne fraction. The radiative contribution for the TCRE has a coefficient of variation of 0.07 and is relatively small compared with the other contributions.

Hence, inter-model differences in the TCRE on decadal to centennial timescales are primarily affected by the climate sensitivity, the fraction of radiative forcing warming the climate system, and by the land uptake of carbon, and only to a lesser extent by the ocean uptake of carbon.

### (b) Contribution of the Southern Ocean

The TCRE is a global metric given by Δ*T*/*I*_em_. A regional contribution to the TCRE might be inferred from the local rise in surface temperature rise divided by the cumulative carbon emission. The model mean of the rise in surface air temperature south of 36°S is 3.3°C compared with a global-mean surface air temperature rise of 4.1°C after 121–140 years. The rise in surface air temperature from south of 36°S accounts for 18% ± 4% of the rise in global-mean surface temperature (table 5), slightly less than the corresponding fraction of global surface area of 22%. However, this estimate ignores the crucial role of the Southern Ocean in sequestering a disproportionate amount of heat and carbon in the climate system, and its effect on climate feedback (table 5).

**Table 5.** Climate variables over the globe and Southern Ocean (model mean and standard deviation) together with the area-weighted fractional contribution south of 36°S to the global mean. Diagnostics for years 121–140 for subsets of 11–21 CMIP6 Earth system models (table 1) following a 1% annual increase in atmospheric CO_2_. The surface area and ocean surface area south of 36°S accounts for 22% and 25.5% of their global surface area and the ocean surface area, respectively. The fractional contribution is provided in terms of the model mean and the standard deviation. Contributions in italics are those that are greater than expected given the surface area contribution.

	surface warming	cumulative ocean heat uptake	cumulative ocean carbon uptake	climate sensitivity	thermal contribution to the TCRE
symbol	ΔT	$Q_{\mathrm{uptake}}$	$\Delta I_{o}$	$\lambda^{-1}$	$-\lambda^{-1}(1-N/\Delta F)$
units	°C	10^22^ J	PgC	K (W m^−2^)^−1^	K (W m^−2^)^−1^
global mean	4.10 ± 0.86	274 ± 41	535 ± 47	−0.83 ± 0.21	0.56 ± 0.12
Southern Ocean mean	3.32 ± 1.09	110 ± 16	235 ± 23	−1.06 ± 0.37	0.72 ± 0.16
fractional contribution south of 36°S to the global mean	18% ± 4%	*40% *± 5%	*44% *± 2%	*28% ± 7%*	*28% ± 3%*
fractional surface area	22%	25.5%	25.5%	22%	22%

The Southern Ocean is significant in affecting the climate response through its contribution to climate sensitivity, from the inverse of the physical climate feedback, *λ*^−1^, accounting for 28% ± 7% of the global-mean climate sensitivity, greater than its proportion of the surface area of 22% (table 5); this area-weighted estimate ignores any additional contribution from the communication of climate feedbacks via a spatial coordination in the feedbacks, referred to as the pattern effect [30]. Accordingly, the Southern Ocean plays an important role in affecting the thermal contribution to the TCRE, Δ*T*/Δ*F* = −*λ*^−1^(1 − *N*/Δ*F*) in (2.13), accounting for 28% ± 3% of its global-mean contribution to the TCRE (table 5).

In summary, the global climate metric, the TCRE, is affected by thermal, radiative and carbon contributions. The inter-model spread in the TCRE is most strongly set by the thermal contribution involving both the effect of climate feedbacks and the fraction of radiative forcing used to increase the heat stored in the planet. The Southern Ocean is important in affecting the global-mean climate sensitivity and the thermal contribution to the TCRE, and so then affects inter-model differences in the TCRE (figure 10; table 4) and the ability to meet associated warming targets.

## 6. Discussion and Conclusion

The Southern Ocean is a unique region of the global ocean in both sequestering anthropogenic heat and carbon via subduction of mode and intermediate waters, and by providing a window to the deep ocean via the upwelling of deep waters. Based on CMIP6 Earth system model projections following an idealized 1% annual increase in atmospheric CO_2_, the surface temperature changes over the Southern Ocean are slightly less than the global-mean response, while the surface carbon responses are comparable to the global mean. For this scenario, the Southern Ocean plays a significant role in the global uptake of anthropogenic carbon and heat, taking up 44%  ± 2% and 40% ± 5%, respectively, of the global ocean uptake of anthropogenic carbon and heat, much greater than its proportion of global ocean surface area of 25.5% using a definition of south of 36°S. This enhanced uptake of anthropogenic carbon and heat exceeds their local ocean storage through a northward transport of anthropogenic carbon and heat to northern ocean basins [12], so that the surface ocean does not equilibrate to the atmospheric conditions and instead air-sea fluxes continue to be strongly directed into the ocean.

### (a) Ocean heat storage

There are large inter-model differences in the Southern Ocean storage of heat, which are not simply explained by the ocean redistribution of heat. Instead the energy balance at the top of the atmosphere needs to be addressed in order to fully understand why Earth system models differ in their storage of heat. Even if the Earth system models experience a similar radiative forcing, the planetary heat uptake differs between Earth system models according to the heat lost to space through the radiative response involving physical climate feedbacks. For the CMIP6 Earth system models, there are inter-model global differences in the physical cloud feedbacks, especially involving shortwave and longwave responses to clouds [32]. For the response over the Southern Ocean, the physical feedbacks involving surface albedo are especially important, a decline in sea ice leading to an enhancement in surface warming [82].

While the vast bulk of the planetary heat input at the top of the atmosphere ultimately passes into the ocean, generally there is not a local equivalence between the planetary heat input at top of the atmosphere and the air-sea heat flux into the ocean due to atmospheric divergences in heat transport. This mismatch is possibly affected by the role of ocean circulation in setting regions of efficient ocean heat uptake, such as the Southern Ocean or equatorial Pacific, where upwelling continually renews the surface layers. Elsewhere, atmospheric convergence of heat acts to stratify the surface ocean and reduce the efficiency of heat uptake by the ocean. Over the Southern Ocean, there are some bands of heat input at the top of the atmosphere partly coinciding with an underlying air-sea heat flux directed into the ocean.

### (b) Ocean carbon storage

The Southern Ocean is important for the sequestration of carbon. There is a broadly consistent response in the ocean climate models to the climate projections [37]. Increasing atmospheric CO_2_ acts to enhance the store of carbon throughout the ocean. Increasing surface temperature and climate feedbacks, though more weakly, act to reduce the ocean store of carbon, involving competing ventilation and biological responses that are particularly large over the Southern Ocean.

### (c) Climate metrics

Global climate metrics, such as the TCRE and the amount of carbon emissions before warming targets are exceeded, are affected by a combination of thermal, radiative and carbon responses. Inter-model differences in the TCRE on decadal to centennial timescales are most strongly affected by inter-model differences in the thermal contribution to the TCRE for the CMIP6 generation of Earth system models [29]. This thermal contribution is determined by the climate sensitivity and the fraction of the radiative forcing warming the climate system. Based on an area-weighting of the climate sensitivity and planetary heat uptake, the climate response over the Southern Ocean contributes typically 28% towards the global-mean thermal contribution to the TCRE, greater than its fraction of global surface area of 22%. This estimate ignores though any additional far-field contribution that the Southern Ocean might make to the pattern of sea surface temperature via cloud feedbacks [88], and hence affecting global climate sensitivity and planetary heat uptake. Through inter-model differences in climate sensitivity and heat uptake, the Southern Ocean sector is also then important in affecting inter-model differences in the TCRE and associated warming targets. The carbon contribution to the TCRE may become more significant on timescales longer than centennial timescales.

### (d) Comparison of our analyses for heat and carbon uptake in the Southern Ocean

Our CMIP6 carbon analyses of the Southern Ocean, accounting for 44% ± 2% of the anthropogenic carbon uptake over the global ocean following a 1% annual rise in atmospheric CO_2_, are very close to CMIP5 historical analyses of the Southern Ocean, accounting for 43%  ± 3% of the anthropogenic CO_2_ uptake by the global ocean [15].

There is a mismatch, however, between our CMIP6 thermal estimates of the Southern Ocean, accounting for 40% ± 5% of the global ocean heat uptake following a 1% annual rise in atmospheric CO_2_, and the much higher estimates from CMIP5 analyses, accounting for 75% ± 22% of the global ocean heat uptake over the historical period [15]. These differences in the thermal response are not accounted for by the slight differences in the definition of the Southern Ocean in both studies, extending either south of 36°S or 30°S; the proportion of Southern Ocean heat uptake only increases slightly to 44% ± 5% of the global ocean heat uptake if the Southern Ocean is defined extending south of 30^o^S (see the electronic supplementary material, figures S1 and S2). Our diagnostics of five CMIP6 models following historical scenarios likewise show a dominant role of the Southern Ocean, accounting for more than 70% or 65% of the global ocean cumulative heat uptake in each model for the periods 1870–1995 or 1870–2010, respectively (see the electronic supplementary material, table S1). Hence, the primary reason for differences in the dominance of the Southern Ocean in global ocean heat uptake is the choice of forcing scenarios: there is a less dominant role of the Southern Ocean in our idealized scenario of a 1% annual increase in atmospheric CO_2_ and a more dominant Southern Ocean role in a historical scenario.

The explanation for the increased role of the Southern Ocean in historical heat uptake is the presence of other radiative forcing agents including aerosols. Historical forcing includes larger hemispheric contrasts in radiative forcing [20] due to natural variability in volcanic and aerosol forcing, which reduces the radiative forcing over the Northern Hemisphere. Consequently, our lower estimate of the fraction of the global ocean heat taken up by the Southern Ocean is due to a more uniform radiative forcing pattern experienced with an increase in atmospheric CO_2_. Our analyses based on idealized scenarios then underemphasize the impact of the Southern Ocean in global ocean heat uptake under historical forcing. However, our analyses provide a useful context as to the relative importance of the Southern Ocean under more evenly distributed radiative forcing, such as may be expected to emerge under future forcing scenarios as CO_2_ forcing becomes more dominant, and aerosol and other anthropogenic forcing are reduced [21].

A major caveat to our study is the reliance on Earth system models with many known deficiencies, including uncertainties in cloud feedbacks, coarseness in ocean resolution and simplicity in the representation of biology. The Southern Ocean in particular remains difficult to accurately resolve in Earth system models, and many of the issues noted in CMIP5 [16], such as general warm surface water biases [17], unrealistic historical sea ice loss [89] and a tendency for mixed layers to shoal and stratify [18] rather than deepen and stratify [90] persist, though are typically reduced, in CMIP6 [78]. There have been some improvements in CMIP6 over CMIP5 in the atmospheric variability, particularly the position and variability of the westerly wind jet [91]. However, there remains an underlying signal to noise problem in how well the Earth system models represent atmospheric variability [92], such as particular atmospheric modes, which will then imprint itself onto the ocean physical and biogeochemical responses. While we accept these caveats into the skill of these models, we are using the projections to gain some insight into the relative importance of different thermal and carbon processes in determining the future climate response to CO_2_ emissions on a centennial timescale.

In summary, the Southern Ocean is disproportionately important in sequestering anthropogenic heat and carbon in the climate system. Much of that anthropogenic heat and carbon is not simply stored locally, but redistributed to northern latitudes. The dominance of the Southern Ocean in affecting the global uptake of heat is affected by the forcing scenario with a smaller fraction of heat taken up in idealized scenarios with more uniform radiative forcing. The thermal response over the Southern Ocean is strongly affected by physical climate feedbacks, involving competing effects from surface albedo and clouds. The Southern Ocean affects a global climate metric, the TCRE, primarily by a thermal contribution, involving the climate sensitivity and ocean heat uptake, and to a lesser extent through a carbon contribution involving the ocean-borne fraction.

## Data Availability

The data used here are from the CMIP6 simulations performed by the various modelling groups and available from the Earth System Grid Federation at the CMIP6 archive (https://esgf-node.llnl.gov/search/cmip6, World Climate Research Programme, 2021). The derived datasets used for the global-mean and Southern Ocean diagnostics are available at https://doi.org/10.5281/zenodo.7562454 [93]. The data are provided in the electronic supplementary material [94].
